# Harnessing the therapeutic effects of nature for chronic Pain: A role for immersive virtual reality? A narrative review

**DOI:** 10.1002/ejp.4727

**Published:** 2024-09-10

**Authors:** Alexander Smith, Kayleigh J. Wyles, Sonia Medina Hernandez, Sophie Clarke, Patricia Schofield, Sam W. Hughes

**Affiliations:** ^1^ School of Psychology, Faculty of Health University of Plymouth Plymouth UK; ^2^ Department of Clinical and Biomedical Sciences, Faculty of Health and Life Sciences, Barrack Road University of Exeter Exeter Devon UK; ^3^ School of Nursing and Midwifery, Faculty of Health University of Plymouth Plymouth UK

## Abstract

**Background and Objective:**

There is a growing interest in the relationship between nature and pain relief. Evidence from environmental psychology, neuroscience and physiology‐based studies point towards analgesic effects of nature being mediated through various cognitive, affective and/or autonomic factors. Being able to harness these therapeutic effects using immersive virtual reality (VR) could help to optimize and improve accessibility of nature‐based environments as part of chronic pain management plans. In this narrative review, we present evidence supporting a new theoretical framework for nature‐based analgesia and suggest ways for applying this through immersive VR.

**Databases and Data Treatment:**

We provide an overview of the evidence on (1) the therapeutic effects of nature on pain, (2) environmental psychology theory that underpins the health benefits of nature, (3) key mechanistic evidence from nature neuroimaging and physiology‐based studies, (4) previous studies that have used VR‐based nature in pain research and (5) how to design effective VR interventions that can be used to integrate nature into immersive 360 environments.

**Results:**

We have demonstrated how environmental psychology, neuroscience and physiology‐based research can be used to form a novel theoretical framework for nature‐based analgesia. Using this framework, we identify how key aspects of nature can act as analgesic and how this can be harnessed using immersive VR.

**Conclusions:**

Through developing this theoretical framework, we have provided a foundation on which to guide future experimental and clinical studies as well as helping to improve the accessibility of nature for chronic pain patients through immersive VR technologies.

**Significance:**

This review article summarizes key multidisciplinary evidence to help understand how nature exerts beneficial effects on pain processing. The use of this theoretical framework alongside advances in immersive VR technologies provides a springboard for future research and can be used to help develop new nature‐based therapeutics using VR.

## 1 INTRODUCTION

Chronic pain is pain that lasts or recurs beyond normal healing time and is defined as pain that persists for more than 3 months (Cohen et al., 2021). Chronic pain syndromes affect up to 20% of the global population and are the single greatest cause of disability across 188 countries, with an estimated 146 million years lost due to disability (Rice et al., 2016). The 11th revision of the International Classification of Diseases (ICD‐11) has recognized chronic pain as either being caused by an underlying condition (i.e. secondary chronic pain) or occurring without any obvious injury or disease (i.e. primary chronic pain; Treede et al., 2015).

The management of chronic pain typically involves a biopsychosocial approach. A combination of pharmacological, psychological and exercise‐based therapies are often prescribed for a range of different primary and secondary chronic pain conditions. However, therapeutic outcomes are often poor with many patients lacking adequate levels of pain relief (Breivik et al., 2006). There is growing need for novel optimized pain therapies that move away from a one‐size‐fits‐all approach to pain management (Bannister & Hughes, 2023). Recent research has shown promising benefits to chronic pain conditions through exposure to nature (Jones & Littzen, 2022) and developing pain therapeutics that can capitalize on the health benefits of being in natural environments could help to reduce the overall global burden of pain (Stanhope et al., 2020). Critically, recent advances in immersive virtual reality (VR) have also provided a new avenue for pain research and treatment (Trost et al., 2021), which could help to improve access to nature‐based pain management programmes. But despite its potential, a current lack of comprehensive knowledge of the theoretical and mechanistic underpinnings of nature‐based analgesia warrants further attention.

Here, we review the evidence and rationale for the use of nature‐based environments for the treatment of chronic pain. A better understanding of the environmental psychology, neuroscience and physiology that underpins nature‐based analgesic responses may enable researchers and clinicians to design and implement optimized pain therapies that aim to leverage the therapeutic properties of being in nature for chronic pain.

## 2 THERAPEUTIC EFFECTS OF NATURE ON PAIN

It is recognized that environmental factors can influence pain and that patients' pain outcomes can be improved if treated in settings that include natural scenes (Malenbaum et al., 2008). There is also growing evidence that utilizing the natural world (i.e. physical features and processes outside of human origin that are perceivable, including the ‘living environment’ of flora and fauna, and non‐living environment of bodies of water, weather and climate, and landscapes displaying geographical characteristics; Hartig et al., 2014) has therapeutic benefits to health and well‐being (Hartig et al., 2014). For example, being in natural environments can help to improve mood, reduce stress (e.g. reduce cortisol, reduce sympathetic/increase parasympathetic tone), reduce depression and anxiety, and improve cognitive function (Hartig et al., 2014). This has been found by engaging with nature (in situ or simulated) in numerous ways: visual (Ulrich, 1984), auditory (Ratcliffe, 2021), olfactory (Bentley et al., 2023) and tactile (Franco et al., 2017). This is particularly relevant for health conditions, such as chronic primary pain, which are often linked to the development of mental health comorbidities, physiological signs of stress and attentional fatigue (Nicholas et al., 2019).

Analgesic benefits through simple visual contact with nature have also been observed in hospital patients experiencing acute pain following surgery. A significant decrease in administered pain relief was required by those with a view over trees than those with a view of brick walls (Ulrich, 1984). Similarly, views of nature murals and natural soundscapes (water or birdcall) reduced pain intensity and anxiety during bronchoscopy procedures in hospital (Diette et al., 2003), and displaying videos of natural environments (waterfalls and mountains) even without sound were observed to strengthen pain tolerance and thresholds in healthy participants (TSE et al., 2002). But what is less clear is the effect of nature on a chronic pain population. There is some evidence to suggest that close proximity to nature is associated with reduced chronic musculoskeletal pain symptoms and factors integral to nature (sunlight, environmental microbiomes, naturescapes and natural soundscapes etc.) could help to reduce the global burden of pain (Stanhope et al., 2020). Chronic pain patients living near greenspaces in urban areas also had a reduced relationship between pain‐related rumination and pain intensity (Wells et al., 2019). Despite there still being a lack of theoretical frameworks to help understand how nature can produce analgesic effects, there is a growing recognition that natural spaces have beneficial effects on chronic pain (Jones & Littzen, 2022), which should also be taken into account during social prescribing and ensuring accessibility to nature as part of urban planning (Lee et al., 2023).

## 3 PSYCHOLOGICAL THEORIES UNDERPINNING THE HEALTH BENEFITS OF NATURE

There are two popular theories from environmental psychology that explain why nature has therapeutic qualities; the Attention Restoration Theory (ART; [Kaplan, 1995]), and the Stress Reduction Theory (SRT; [Ulrich et al., 1991]). Both theories posit that specific environmental stimuli have beneficial effects on one's psychophysiological health. However, neither theory explicitly explains the mechanisms of nature as an analgesic (Jones & Littzen, 2022), meaning their specific application for pain contexts remains to be fully explored.

The ART theory proposes that the ability to concentrate or focus our attention on specific tasks or activities is limited. According to this theory, when we continuously use this directed attention for a long period, we start feeling fatigued (i.e. directed attention fatigue; [Basu et al., 2019]). Kaplan (1995) argues for directed attention to regenerate, an environment needs to have restorative properties that encourage us to instead utilize our effortless attention (an infinite resource):

A sense of being away from everyday pressures or stressful contexts, either physically or conceptually;

A good person‐environment compatibility to facilitate an intended action or goal;

Sufficient extent, scope, and coherence to support visual engagement and exploration;

Qualities, features or patterns which are fascinating enough to capture attention effortlessly.

These features are posited to occur frequently in nature; for instance, nature is often away from everyday demands, can facilitate a range of actions like running or playing on the beach; is stimulating with its variety of flora and fauna; and the trickle of water in a brook, birdsong, the rustle of leaves in the wind all allow ‘soft’ fascination which involuntarily captures one's attention, but still allows for mind wandering (Ohly et al., 2016). The ART distinguishes between soft and hard fascination, wherein the latter occupies the mind completely—sometimes forcefully, as in the case of survival‐related stimuli—and offers no space for reflection (e.g. watching a murder mystery programme or spotting a snake; [Basu et al., 2019]). Thus, natural stimuli supporting ‘soft’ fascination contrast those ‘harder’ stimuli encountered in more urban environments (e.g. advertisements, traffic) which are deemed to require conscious effort to filter and drain directed attention (Berman et al., 2008).

Importantly, suitable compatibility between an individual and the environment is necessary to facilitate restoration; incompatibility can exacerbate directed attention fatigue (Herzog et al., 2010). For the chronic pain community, spaces otherwise optimally restorative to a healthy population could be incompatible due to inaccessibility, navigability or physical traversal difficulties (Perry et al., 2021). Differences between individual physical ability, experiences and goals, could impact one's compatibility with a typically restorative environment: Being in a forest may provoke fear of being injured or getting lost, but be fascinating and tranquil for others (Gatersleben & Andrews, 2013).

Ultimately, the ART primarily describes benefits to cognition or allowing ‘mental housekeeping’ of lingering thoughts and ruminations, thereby also improving mental well‐being (Basu et al., 2019). Given that chronic pain is often associated with depleted attentional resources and reduced performance in cognitive tasks (Eccleston, 1995), it is possible that nature can exert beneficial effects on pain outcomes (e.g. pain intensity or pain‐related rumination) through enhanced attentional capacity, reduced directed attentional fatigue as well as enhanced focus and cognitive control (Wells et al., 2019).

On the other hand, the SRT poses that nature could help to reduce pain as it facilitates the reduction of stress because it embodies qualities considered safe and desirable from an evolutionary perspective (e.g. water, trees and lack of threats [Ulrich et al., 1991]). Thus, the SRT describes features of natural environments as fuelling positive affect, thus combatting negative feelings, stress and regenerating one's adaptive resilience (Ulrich et al., 1991). The core of SRT posits that restorative environments are appraised as such based on our evolutionary adaptive responses regarding subjective threats; thereby, dangerous, overstimulating and/or evolutionary new environments (i.e. we have only been living in urban settlements like towns and cities relatively recently, thus do not have an evolutionary response for this environment) are not restorative, but serene, safe and calm areas that we evolved in thousands of years ago are (Ulrich et al., 1991). To that end, reductions to stress‐related physiological measures (e.g. galvanic skin response, systolic blood pressure and muscle tension) have been observed in participants experimentally stressed with a black and white workplace accident video (i.e. injuries within a woodworking shop which show simulated blood and mutilation), who were then exposed to nature, along with self‐reported feelings of fear, positive affect, anger, attentiveness and sadness (Ulrich et al., 1991).

Taken together, both ART and SRT can help to explain the beneficial effects of nature on pain and pain‐related outcomes. These psychological theories suggest that pain relief could be a result of improved cognitive control (ART) coupled with the numerous affective and physiological changes that are associated with being in nature (SRT). To further apply these current theoretical models (e.g. ART and SRT), more work is needed to apply them directly to pain‐related experiences and behaviours, identify the key features of nature that encourages these health benefits and to integrate with neural mechanisms and pain‐related behaviour.

## 4 CHANGES IN BRAIN ACTIVATION DURING EXPOSURE TO NATURE

Functional neuroimaging studies have helped to shed some light on the neural mechanisms that underpin the therapeutic effects of nature, which show overlap with key differences in affective brain activity seen during chronic pain. A recent study has demonstrated that resting state functional connectivity between the amygdala and the inferior parietal lobe mediates the relationship between catastrophizing and altered threat‐safety discrimination learning during persistent pain states (Timmers et al., 2022). Within healthy cohorts, exposure to nature, but not urban environments, can lead to a reduction in activity in the amygdala during a standardized fearful face task (Sudimac et al., 2022). Taken together, these studies show how chronic pain can influence corticolimbic brain regions involved in threat‐safety learning and suggests that nature may promote effective emotional regulation during stress‐evoking situations. This overlap between pain‐generating mechanisms seen during chronic pain and regions influenced by exposure to nature warrants further investigation.

Bratman et al. (2015) also showed that following a walk in nature, individuals exhibited reductions in self‐reported rumination as well as blood perfusion (a marker of resting state brain activity) in the subgenual prefrontal cortex, a key node for regulation of emotional responses to pain (Petrovic and Ingvar 2002). Negative relationship between increased positive affect and prefrontal cortex activity decreases was also found following exposure of urban green spaces (Tost et al., 2019). Furthermore, prefrontal cortex activity reductions following shinrin‐yoku (i.e. forest bathing) have also been observed alongside reductions in salivary cortisol, a physiological maker of stress (Park et al., 2007). Taken together, functional neuroimaging results suggest that, compared to exposure to urban environments, nature can trigger activation in brain regions known to be responsible for top‐down regulation of emotional responses to stressors, while simultaneously reducing activity in areas associated with fight/flight/freeze reactions (Kim et al., 2010). Critically, these changes seen during exposure to nature appear to overlap with corticolimbic brain regions involved in the maladaptive regulation of pain‐related afference which are thought to contribute to the development of chronic pain states (Mansour et al., 2014; Yang & Chang, 2019).

## 5 AUTONOMIC CHANGES DURING EXPOSURE TO NATURE

It is recognized that exposure to nature reduces stress and SRT is one of the key theories underpinning the therapeutic benefits of nature. The autonomic nervous system regulates stress responses through balance of activity in the sympathetic (fight/flight/freeze in the presence of stress) and parasympathetic (rest/digest in the absence of stress) systems. The vagus nerve is a fundamental component of the parasympathetic system, and therefore, vagal tone may serve as an objective indicator of stress levels (Porges, 1995). Heart rate variability is predominantly dependent on vagal modulation during rest and is a key, easy to obtain physiological measure that reflects vagal cardiac effects (Sztajzel, 2004; Thomas Bianca et al., 2019). Specifically, high frequency heart rate variability has become a widely accepted marker of parasympathetic activity, with increases being frequently linked to stress reduction (Kim et al., 2018).

A growing number of studies have demonstrated increases in high frequency heart rate variability following exposure to nature, indicating enhanced parasympathetic tone (Brown et al., 2013; Gladwell et al., 2012, 2016; Lee et al., 2014). Overall, recent systematic reviews have concluded that virtual exposure to nature can elicit increases in parasympathetic tone (Jo et al., 2019), in line with reductions in self‐reported measures of negative mood and anxiety (Gaertner et al., 2023; Jo et al., 2019). In contrast to these studies, it has also been shown in one study that heart rate variability can decrease after prolonged exposure to nature, despite reduction in self‐reported stress, with the authors proposing that this could reflect enhanced arousal, excitement and positive mood in the natural environment (Scott et al., 2021). Further studies are needed to fully elucidate the effects of nature on autonomic responses and how these relate to analgesic mechanisms in the brain.

Many of the brain regions that control autonomic output are also involved in pain perception and modulation of pain responses, including the insular cortex, anterior cingulate cortex, amygdala and periaqueductal grey (Benarroch, 2006; Cerritelli et al., 2021). Furthermore, maladaptive autonomic responses following painful stimulation, measured through systolic blood pressure and high frequency heart rate variability respectively, have shown to relate to less efficient descending pain modulatory capacity, evidenced by reduced inhibitory responses during a conditioned pain modulation paradigm in healthy (Makovac et al., 2021) and chronic pain cohorts (Chalaye et al., 2014). Across multiple studies, higher parasympathetic tone is correlated with higher capacity for pain inhibition (Forte et al., 2022). In contrast, decreased high frequency heart rate variability (i.e. indicating decreased parasympathetic activity) has been proposed as a potential biomarker for chronic pain (Tracy et al., 2016). Hence, parasympathetic tone increases induced by nature may be a further mechanism by which nature induces analgesia.

## 6 INTRODUCING A THEORETICAL FRAMEWORK FOR NATURE‐BASED ANALGESIA

**FIGURE 1:**
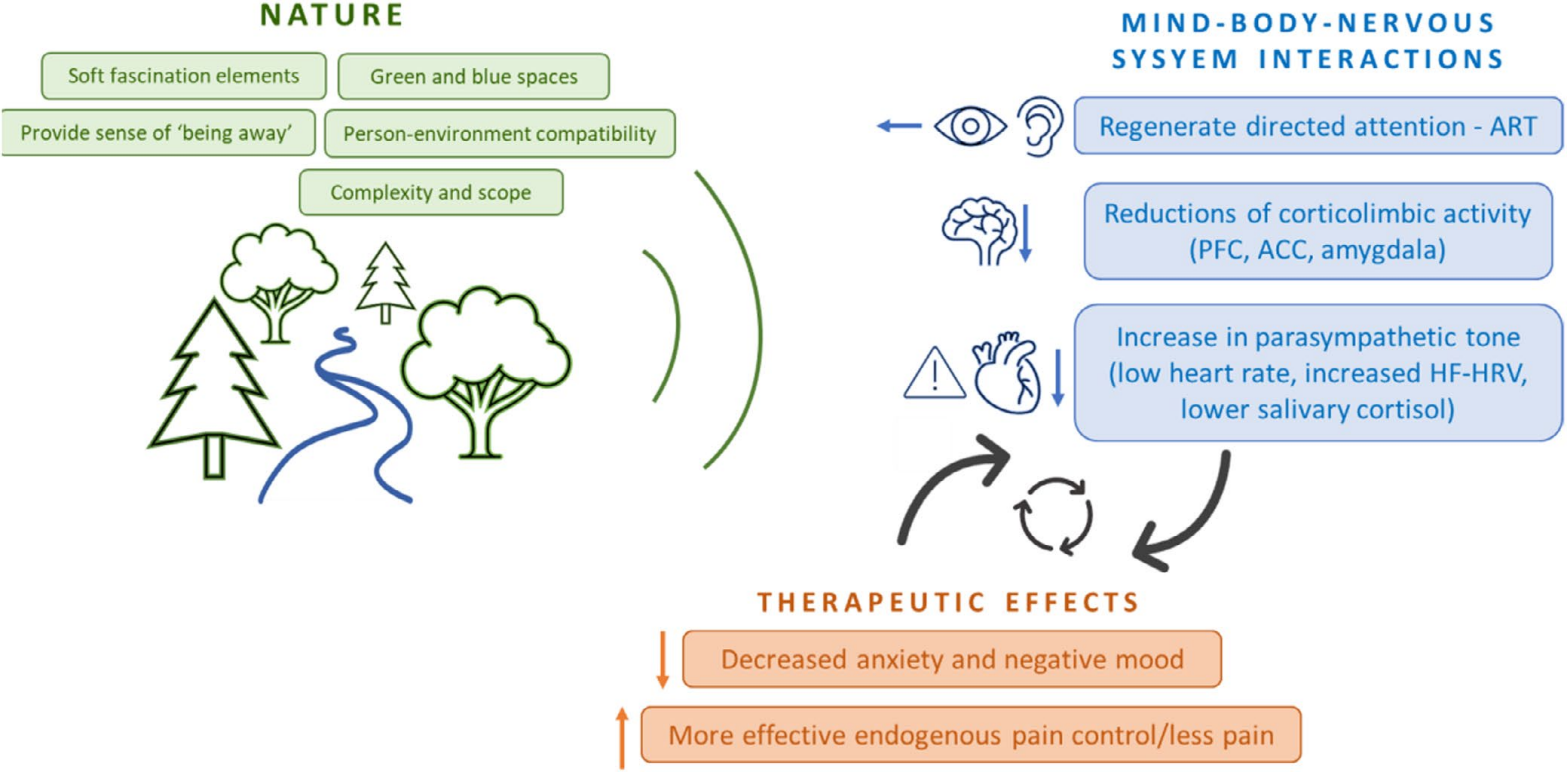
Theoretical framework for nature‐based analgesia. Natural environments should meet a number of key features: (i) Fulfil the criteria in the figure sense of being away (e.g. distance from stressors; immersion and presence); (ii) be compatible with the individual's needs and desires (e.g. an appropriate fit for the activity or goal); (iii) contain extent or scope for exploration and visual complexity (e.g. perceived biodiversity, paths veering away); (iv) be inherently ‘softly’ fascinating and engaging without consuming too much attention (e.g. birdsong, water flowing); (v) be associated with safety with a lack of threats (e.g. lack of visibility for danger) such as reduction of stress, restoration of directed attention and overall improved mental well‐being. These key features of natural environments can result in activation of brain regions involved in mediating stress, cognition, mood and autonomic function; all of which play an important role in pain processing. ACC, anterior cingulate cortex; ART, attention restoration theory; HF‐HRV, high frequency heart rate variability; PFC, prefrontal cortex; SRT, stress reduction theory.

The individual psychological, neurophysiological and physiological theories and evidence presented so far can be pieced together, to construct a comprehensive theoretical model that can be used to design future studies to help explain the mechanisms underpinning nature‐based analgesia (Figure 1). We propose that, in a nuanced process, exposure to nature, with its distinctive restorative features, initiates a cascade of psychological and physiological responses. Being in nature prompts a reduction in corticolimbic activity, particularly within brain regions associated with the processing of emotional aspects of pain. This suggests a mitigating effect on the impact of negative affective dimensions of the pain experience. Furthermore, a reduction in stress may facilitate an increase in parasympathetic tone, promoting a heightened state of relaxation. This result might not only enable individuals to further engage with the environment, but it may also facilitate the redirection of attention away from pain, in line with ART. These intricate mind–brain–body interactions may perpetuate a cyclical process, where reductions of stress, autonomic regulation and enhancement of relaxation and cognitive function reciprocally influence each other, ultimately leading to a higher sense of well‐being and reduced pain in the individual. Critically, this theoretical framework allows for future mechanistic studies to be designed to test specific hypotheses related to the effects of nature on these key mind–body–nervous interactions and behavioural outcomes. For example, within this theoretical framework, future research may explore the potential of nature to cultivate a more adaptive interaction between autonomic regulation, cognitive control and endogenous pain control systems.

## 7 HARNESSING THE THERAPEUTIC EFFECTS OF NATURE WITH IMMERSIVE VIRTUAL REALITY (VR)

Given the psychological and physiological health benefits of nature, being able to leverage these therapeutic effects for the amelioration of chronic pain symptoms using immersive VR (i.e. the perception of being physically present in a non‐physical world) could lead to new pain management approaches. Adequate accessibility to being in situ with nature presents a problem for the chronic pain community, especially for elderly individuals who are already physically vulnerable (Li et al., 2021; Patuano, 2020). Chronic pain patients are sometimes limited in their ability to access, and potentially physically navigate, natural environments, particularly in those who live in residential urban areas. Thus, in certain cases, it may be beneficial to adapt the stimuli experienced in nature to the virtual world through advances in immersive VR technologies, while also taking into consideration suitable compatibility and preferences between an individual and the environment. Nature‐based VR has been shown to have similar positive effects on restorativeness (Browning et al., 2019) and a similar sense of presence (Chirico & Gaggioli, 2019) compared to actual nature. It therefore has the potential to be used in cases where patients are unable to leave the home or to help further augment real‐world restorative experiences in nature. The flexibility of virtual environments to be tailored to specific needs also opens up the opportunity to use VR to deliver a combination of different therapeutic interventions, such as pain education (McConnell et al., 2024; Pandrangi et al., 2019), alongside the restorative nature‐based experiences to help improve overall pain management.

There has been a rapid rise in the use of VR in pain management (Trost et al., 2021) with a growing number of studies that have used natural environments within VR as a therapeutic intervention in clinical pain conditions (Berdejo‐Espinola et al., 2024). For example, the use of VR nature scenes (e.g. oceans and forests) have been shown to have beneficial effects on pain and pain‐related symptoms in cancer patients (Chin et al., 2022; Kelleher et al., 2022). Walking through a forest (via a virtual walking video clip using video glasses) has also shown potential benefits for pain, fear of movement (kinesiophobia) and disability in individuals with chronic low‐back pain (Yelvar et al., 2017). Importantly, using immersive VR natural landscapes can produce stronger analgesic effects compared to non‐immersive and imagined landscapes in both chronic pain patients and healthy participants (Tesarz et al., 2024). It is therefore important to consider the potential for using immersive 360º VR to deliver nature experiences as part of future clinical trials for patient groups who have restricted access to nature.

However, a major methodological issue in the field of VR for pain management is disentangling the effects of pure distraction from other mechanisms specific to the immersive environments being experienced (e.g. nature specific effects). A number of nature VR studies have used gamified tasks, or otherwise deliberately induced distraction, which is a commonly accepted theory for explaining the effectiveness of generic VR‐based analgesia (Ahmadpour et al., 2019; Malloy & Milling, 2010). While using gamification elements in VR settings that mimic nature can help reduce pain intensity levels, the distraction caused by playing a game could interfere with the mental recovery processes suggested by the attention restoration theory. This is because it relies on intense focus (hard fascination) rather than the gentle engagement (soft fascination) typically promoted by nature. For soft fascination to function in terms of psychological restoration (and thus fully maximizing the health benefits of nature in VR), the environments must be sufficiently engaging and restorative to capture attention effortlessly, while allowing adequate cognitive resources for self‐reflection and directed attention restoration (Basu et al., 2019). It is therefore important to design VR nature environments with clear restorative features that can be incorporated into well thought out experimental designs and measured using psychometric questionnaires (e.g. the perceived restorativeness scale; Hartig et al., 1997) alongside adequate controls (e.g. attentional tasks, non‐nature scenes). Well‐designed nature VR investigations in combination with neurophysiological, neuroimaging, psychophysical and psychometric techniques will help to disentangle the effects of nature VR and pure distraction‐based VR in a controlled laboratory‐based setting. Our new theoretical model encourages future research to test specific causal relationships between specific features of nature (e.g. levels of fascination) and the key cognitive, affective and autonomic mechanisms that are thought to be involved in producing the analgesic effects.

## 8 OPTIMIZING THE DESIGN OF NATURE‐BASED VR ENVIRONMENTS

It is important to note that for VR‐based natural environments to be ecologically valid, they must provide a degree of immersion and presence alongside restorative effects that resembles being in a real‐world environment (Kothgassner & Felnhofer, 2020). Immersive VR is defined based on the degree of sensory engagement with the virtual world (Alqahtani et al., 2017). It is an objective quality and is related to technical parameters of the VR system used (Cummings & Bailenson, 2016; Grassini & Laumann, 2020). Accordingly, VR can be divided into *non‐immersive* (e.g. a 2D screen), *semi‐immersive*, where users are able to navigate a more engaging 3D virtual world, but without real physical movements within it (e.g. driving simulators, dome screens) *and fully immersive*, which ensures much more realistic experience within the virtual world with six degrees of freedom, allowing the user to move in all directions and interact with the environment (e.g. exposure to real‐life situations or navigation through real places) (Tcha‐Tokey et al., 2016).

It is also possible to deepen the immersive experience using high‐resolution, 360º videos or photographs to enhance the sense of presence and restorativeness of the natural environments (Newman et al., 2022). Designing interventions which can also incorporate spatial audio that replicates the natural sounds of the environment, such as bird calls, wind rustling through trees and water flowing can all further contribute to the levels of realism and immersion (Ratcliffe, 2021). The development of new user‐friendly nature‐based VR experiences should also consider whether users can interact with the environment, such choosing paths to walk or interacting with objects which can help to improve engagement and personalize the experience. It is also recognized that a criticism of using virtual experiences is that the social connections associated with being in nature with others is lost. However, recent advances have made multi‐user VR experiences a possibility (Xu et al., 2024), which could help to improve levels of social interaction within nature‐based VR experiences.

Different levels of immersion and realism lead to particular levels of presence, defined as the subjective illusion of being part of the virtual environment, rather than the real environment (Slater, 2018). This is essential for effective VR interventions, as it involves the correspondence between proprioceptive states and sensory inputs (Sanchez‐Vives & Slater, 2005). It is critical to acquire an understanding of individual levels of presence during nature‐based VR environments alongside the perceived restorativeness of the environment to appropriately quantify the experience of nature within a VR environment. This can be done using questionnaires, such as Witmer and Singer's Presence Questionnaire or Slater‐Usoh‐Steed Questionnaire alongside the Perceived Restorativeness Scale within the actual VR environment or shortly following the end of the experience (Schwind et al., 2019).

It is also important to understand the influence of nausea or discomfort related to the VR experience. A number of factors relating to the hardware, content and human factors (e.g. age, gender and prior experience with VR) can all have a large impact on the VR experience (Chang et al., 2020). The most commonly used questionnaire used to measure severity of VR cyber sickness is the Simulator Sickness Questionnaire, which can provide detailed information on nausea, oculomotor and disorientation symptoms.

## 9 CONCLUSION

A novel theoretical framework for nature‐based self‐management of chronic pain has been proposed, which brings together environmental psychology theory (e.g. ART, SRT), neuroscience (e.g. corticolimbic and top‐down pain processing) and physiology (e.g. autonomic function). We also highlight the potential and future considerations for integrating nature‐based environments within immersive VR technologies. Critically, research in this area will aid the understanding of how key aspects of nature can act as analgesic and could potentially target key pain‐generating mechanisms (e.g. dysfunctional pain processing and modulation) in chronic pain patients.

## FUNDING INFORMATION

A. Smith's (and his supervisors, S Hughes, K. Wyles and P. Schofield) contribution is supported through a PhD studentship awarded by the Faculty of Health of the University of Plymouth. S. Hughes received funding through an Academy of Medical Sciences Springboard grant (SBF007\100,108) and the Engineering and Physical Sciences Research Council (EPSRC) funded Chronic Pain Neurotechnology Network+ (EP/W03509X/1) which also helped to support this work.
